# Electroacupuncture for Bladder Function Recovery in Patients Undergoing Spinal Anesthesia

**DOI:** 10.1155/2014/892619

**Published:** 2014-12-24

**Authors:** Yinqiu Gao, Xinyao Zhou, Xichen Dong, Qing Jia, Shen Xie, Ran Pang

**Affiliations:** ^1^Division of Anesthesia, Guang An Men Hospital, China Academy of Chinese Medical Sciences, Beijing 100053, China; ^2^Division of Internal Medicine, Guang An Men Hospital, China Academy of Chinese Medical Sciences, Beijing 100053, China; ^3^Division of Urology, Guang An Men Hospital, China Academy of Chinese Medical Sciences, No. 5 Bei Xian Ge Street, Xicheng District, Beijing 100053, China

## Abstract

*Purpose*. To determine the efficacy of electroacupuncture on recovering postanesthetic bladder function. *Materials and Methods*. Sixty-one patients undergoing spinal anaesthesia were recruited and allocated into electroacupuncture or control group randomly. Patients in electroacupuncture group received electroacupuncture therapy whereas ones in control group were not given any intervention. Primary endpoint was incidence of bladder overdistension and postoperative urinary retention. Secondary endpoints included time to spontaneous micturition, voided volume, and adverse events. *Results*. All patients (31 in electroacupuncture group and 30 in control group) completed the evaluation. During postoperative follow-up, patients in electroacupuncture group presented a significant lower proportion of bladder overdistension than counterparts in control group (16.1% versus 53.3%, *P* < 0.01). However, no significant difference was found in incidence of postoperative urinary retention between the two groups (0% versus 6.7%, *P* > 0.05). Furthermore, a shorter time to spontaneous micturition was found in electroacupuncture group compared to control group (228 min versus 313 min, *P* < 0.001), whereas urine volume and adverse events had no significant difference between the two groups. *Conclusions*. Electroacupuncture reduced the proportion of bladder overdistension and shortened the time to spontaneous micturition in patients undergoing spinal anesthesia. Electroacupuncture may be a therapeutic strategy for postanesthetic bladder dysfunction.

## 1. Introduction

Spinal anesthesia is the simplest and most reliable technique in regional anesthesia. It is widely used for lower abdominal, pelvic, perineal, and lower extremity surgery [1]. Compared to general anesthesia, spinal anesthesia has numerous advantages including rapid onset, lower patient discomfort during the procedure, and decreased need for postoperative analgesia [2, 3]. However, spinal anesthesia is more likely to result in detrusor dysfunction due to blocking the afferent and efferent limbs of the micturition reflex and may subsequently lead to bladder overdistension (BOD). Once bladder is sufficiently overdistended, detrusor contractility remains impaired, which can develop postoperative urinary retention (PUR) [4]. Although urethral catheterization has been believed as the standard care for PUR, catheter-associated urinary tract infection not only lengthens the hospital stay but also increases the mortality [5]. To prevent the PUR, some therapeutic attempts, such as restrictive fluid regimens [6], ingestion of caffeine [7], and administration of *α* blockers [8, 9], have been used in clinical practice. Unfortunately, these strategies cannot meet all the clinical needs due to their limited applying fields. Studies have shown that fluid restriction cannot lower the incidence of PUR in low-risk surgery patients [10, 11]. Caffeine is not suitable for patients with overactive bladder or urinary incontinence, because it may worsen these symptoms [12, 13]. Administration of *α* blockers may lead to severe hypotension for patients undergoing spinal anesthesia because blood pressure lowered by spinal anesthesia [14] may be further reduced by *α* blockers. Therefore, it is necessary to develop a new therapy to facilitate the recovery of postanesthetic bladder function.

Our study aimed to evaluate the effect of electroacupuncture on recovering bladder function in patients undergoing spinal anesthesia.

## 2. Methods

### 2.1. Study Population

Patients undergoing spinal anesthesia due to arthroscopic knee surgery were recruited from Guang An Men hospital, China Academy of Chinese Medical Sciences between January 2010 and December 2012. Inclusion criteria for the study were patients with American Society of Anesthesia (ASA) Physical Status I or II and age ranging from 18 to 65 years. Exclusion criteria were patients with emergency surgery, history of bladder outlet obstruction, urinary tract infection, spinal or neurological disorders, and coagulation disorders.

The study protocol was approved by the institutional review board in Guang An Men Hospital and it was designed according to the Declaration of Helsinki, the International Conference on Harmonisation. All study participants provided written informed consent.

### 2.2. Study Design

Patients who met the protocol eligibility criteria were allocated into electroacupuncture or control group randomly, using a random number generator. Spinal anesthesia for all the patients was performed by the same anesthetist. When the level of sensory block regressed to T10 segment, which was checked by pin-prick test, patients in electroacupuncture group received electroacupuncture therapy, whereas ones in control group were not given any intervention. Postoperative bladder volume was monitored by ultrasonography at the end of operation and every subsequent hour until spontaneous micturition. The BOD was defined if a patient's bladder volume was more than 400 mL before spontaneous micturition. Once the bladder volume was more than 600 mL, PUR was considered and an indwelling urethral catheterization was performed to prevent persistent bladder dysfunction [4]. Primary endpoint was the incidence of BOD and PUR, which were calculated in each group and compared to assess the effect of electroacupuncture. The time to spontaneous micturition after spinal anesthesia, urine volume, and adverse events, as secondary endpoints, were recorded and analyzed. Additionally, the extra analgesia which patients administered and the amount of intravenous fluids before spontaneous micturition in each group were also recorded.

### 2.3. Spinal Anesthesia

Patient was positioned with lateral decubitus and spinal anesthesia was performed at L3/L4 interspace with a Quincke 22-gauge needle using hyperbaric tetracaine (0.33%) 10 mg. Hyperbaric tetracaine was composed of tetracaine 1 mL (10 mg), ephedrine 1 mL (30 mg), and 10% glucose injection (1 mL). The tetracaine injection speed was 1 mL per 5 seconds. The maximum level of sensory block was obtained between T6 and T8 segments.

### 2.4. Electroacupuncture Intervention

The electroacupuncture therapy was performed by the same acupuncturist in operation room. Sterile disposable, stainless filiform acupuncture needles, 0.3 mm in diameter and 50 mm in length (HWATO, Suzhou Medical Appliance Factory, Suzhou, China), were used. The selected acupoints included CV3 (Zhong Ji), CV4 (Guan Yuan), and bilateral ST29 (Gui Lai). After the acupuncture needles were inserted into acupoints, Han's acupoint nerve stimulator (model HANS 200E, Beijing Huayun Ante technology Co. Ltd., China) was connected to the handles of acupuncture needles to provide the electrical stimulation for 30 minutes. A low-frequency (2 Hz) continuous wave stimulation was applied.

### 2.5. Data Analyses

Statistical analyses were performed using the SPSS software package for Windows version 19.0 (SPSS Inc., IL, USA). Continuous variables were expressed as means ± standard deviation, while categorical variables were expressed as percentage and frequency. The occurrence rates of BOD and PUR were compared by Pearson's Chi-square test between two groups. Shapiro-Wilk* W* test was used to detect if the data fitted normal distribution in each group. The time to spontaneous micturition, urine volume, and amount of intravenous fluids between the two groups were compared by two-sample* t*-test if the data followed normal distribution; otherwise, Mann-Whitney* U* test was used. The frequency of adverse events was analyzed between the two groups using Fisher exact test. All reported *P* values were two-sided, and *P* < 0.05 was considered statistically significant.

## 3. Results

A total of 61 patients (31 in electroacupuncture group and 30 in control group) were recruited in our study.  Table 1 shows the patients' demographic and baseline (sensory level receded to T10 segment) measurements. No significant difference was found between the two groups (*P* > 0.05). During the procedure, patients' pain was controlled well and no extra analgesia was administered in either group.

During postoperative follow-up, electroacupuncture group showed a significant lower incidence of BOD compared with control group (16.1% versus 53.3%, *P* < 0.01). Of patients with BOD, two patients (36-year-old male and 50-year-old female) in control group developed PUR and were treated with indwelling urethral catheterization, while no patients experienced PUR in electroacupuncture group. There was no significant difference in incidence of PUR between the two groups (0% versus 6.7%, *P* > 0.05). Furthermore, patients in electroacupuncture group presented a shorter time to spontaneous micturition compared to counterparts in control group (*P* < 0.001), whereas no significant difference was found in the urine volume and infusion volume between the two groups (Table 2). Additionally, two and six patients complained of the feeling of incomplete emptying until postoperative day one in electroacupuncture and control group, respectively. The frequency of this adverse event did not show significant difference between the two groups (6.5% versus 21.4%, *P* > 0.05).

In terms of different age group, the younger patients (≤50 years) had a shorter time to spontaneous micturition than the older ones (>50 years) in electroacupuncture group, while no significant difference was found in control group (Table 3). Moreover, younger and older age group presented similar percentage in patients with BOD in electroacupuncture group (12.5% versus 17.4%) and control group (50% versus 55%), respectively. In addition, no significant difference was found between males and females in either group (Table 4).

## 4. Discussion

The main finding of this study is that electroacupuncture can reduce the occurrence rate of BOD and shorten the time to spontaneous micturition in patients undergoing spinal anesthesia.

Disturbance of bladder function is a main issue for patients undergoing spinal anesthesia, which may develop BOD and PUR. The definition of BOD and PUR varies in different studies. Some studies focused on ultrasound assessment [15, 16], while others relied on the clinical examination [17, 18]. In order to ensure accuracy of diagnosis, our study used ultrasound to assess bladder volume. Studies have shown that ultrasound can provide an accuracy of 94% for a predicted bladder volume which is more than 100 mL [19] and only has a measurement error of less than 15 mL [20]. On the other hand, the normal bladder volume remains controversial, ranging from 300 to 600 mL. Although a study showed that bladder volume between 400 and 600 mL is normal [15], 13% patients with bladder volume of more than 400 mL required catheterization after undergoing spinal anesthesia [21]. Urodynamic study revealed that individuals reported bladder distension when their bladder volume reached 400 mL [22]. Moreover, catheterization is recommended for postoperative patients whose bladder volume is more than 600 mL [4]. After taking account of the urodynamic finding and the expert recommendation, we used 400 mL and 600 mL as bladder volume threshold for bladder BOD and PUR, respectively.

The incidence of PUR in general surgical population is about 4% [23] and varies according to the type of surgery. In our study, the occurrence rate of PUR in control group was 6.7% which is lower than the reported 21.1% to 55% [24, 25] in knee surgery. The main reason probably comes from the different patient characteristics and definition of PUR. Our study excluded patients with bladder outlet obstruction, but those studies did not. As is reported, preexisting bladder outlet obstruction is important risk factor for PUR [26]. The various diagnostic criteria of PUR also contribute to the difference in outcome between our study and others.

A study showed that the level of sensory block took seven to eight hours to regress to S3 segment, which allowed patient to urinate spontaneously, after spinal injection of hyperbaric bupivacaine or tetracaine [27]. Our study showed a less time to spontaneous micturition, which was approximately five hours in control group. The difference in recovery time of micturition mainly resulted from the different dose of local anesthetic in spinal anesthesia. In our study, a moderate dose of tetracaine (10 mg) was adopted, while a high dose of tetracaine (15 mg) was used in that study. Another important reason is that a different time point (level of sensory block regressed to T10 segment) was used as baseline to measure the time to spontaneous micturition in our study. Because the level of sensory block varied in different individuals, our study used the time point to minimize measurement bias.

Some studies showed that age and gender were the essential factors for recovery of postanesthetic bladder function. Patients over 50 years and male are more likely to experience BOD and develop PUR after undergoing spinal anesthesia [16, 23, 28]. In our study, the time to spontaneous micturition, urine volume, and the proportion of BOD did not vary by gender or age in control group. The possible reason is that our study ruled out patients with bladder outlet obstruction which always occur in aging or male population. Interestingly, we found that younger patients got faster recovery in bladder function than older ones after they received electroacupuncture therapy. The reason might be that age-related progressive neuronal degeneration delayed the bladder function recovery, which reduced the nerve sensitivity to electroacupuncture therapy.

Acupuncture, as a component of traditional Chinese medicine, has been used for various urological diseases and has gained acceptance among urologists [29]. Some studies have shown its effectiveness on benign prostatic hyperplasia [30], nocturnal enuresis [31], premature ejaculation [32], and urinary incontinence [33, 34]. In our study, we found that electroacupuncture could reduce occurrence rate of BOD and shorten the time to spontaneous micturition. The possible mechanism is the modulation of acupuncture on detrusor contraction. An experimental study demonstrated that needling CV4 (Guan Yuan) could increase the frequency of detrusor contraction in rat model of urinary retention [35] and another study showed that needling CV3 (Zhong Ji) could regulate the vesical pressure in rabbit [36]. It was shown in a clinical study that needling ST29 (Gui Lai) could increase the average urine flow and reduce the postvoid residual urine volume in patients undergoing radical hysterectomy [37]. On the other hand, analgesic effect of acupuncture may also contribute to patients' spontaneous micturition which is disturbed by postoperative pain [38]. Some studies have confirmed the role of acupuncture in postoperative pain management [39–41]. The potential mechanism includes inhibition on conduction of pain signals, release of endorphin, activation of analgesic system, and central modulation [42].

To confirm therapeutic effect of electroacupuncture, blank control was used in our study. Although placebo control serves as the golden standard in clinical trials, no standard approach for acupuncture placebo has been published. Sham acupuncture is a control strategy which has been used in clinical trials. However, a systematic review implies that sham acupuncture may have equal therapeutic effect to real acupuncture [43]. Furthermore, patients with experience of acupuncture treatment can distinguish between real and sham acupuncture [44], which may introduce bias to acupuncture study. Therefore, sham acupuncture was not used as control in our study.

The important limitation of this study includes small sample size and nonblind design. Another limitation is lack of the urodynamic evaluation for bladder function. However, it would cause patients' discomfort and is also a burden for both patients and the health insurance.

## 5. Conclusions

Findings from this study suggest that electroacupuncture reduced the incidence of BOD and shortened the time to spontaneous micturition in patients undergoing spinal anesthesia. Electroacupuncture might be a therapeutic strategy for bladder dysfunction secondary to spinal anesthesia.

## Figures and Tables

**Table 1:** Patient characteristics and measurements at baseline.

	EA group	Control group
Number	31	30
Age (years)	55 ± 6	53 ± 11
Gender		
Male	10	9
Female	21	21
Height (cm)	164 ± 8	166 ± 8
Weight (kg)	69 ± 11	68 ± 9
SBP (mmHg)	115 ± 12	114 ± 13
DBP (mmHg)	66 ± 16	68 ± 11
MAP (mmHg)	83 ± 14	83 ± 10
HR	72 ± 8	72 ± 9
Duration of surgery (min)	83.4 ± 17	85.5 ± 16.4

Values are given as mean ± standard deviation. EA: electroacupuncture; SBP: systolic blood pressure; DBP: diastolic blood pressure; MAP: mean arterial pressure; HR: heart rate.

**Table 2:** Time to spontaneous micturition, urine volume, and infusion volume in the two groups.

	EA group	Control group
*N*	31	28
Time to SM (min)^a^	228 ± 78	313 ± 91^*^
Urine volume (mL)^b^	339 ± 109	361 ± 179
Infusion volume (mL)^b^	1631 ± 270	1673 ± 303

Values are given as mean ± standard deviation. ^a^Difference was analyzed by two-sample *t*-test between the two groups; ^b^Difference was analyzed by Mann-Whitney *U* test between the two groups.  ^*^ *P* < 0.001 versus EA group. EA: electroacupuncture; SM: spontaneous micturition.

**Table 3:** Time to spontaneous micturition, urine volume, and infusion volume in different age group.

	EA group		Control group	
	Younger age group	Older age group	Younger age group	Older age group
	(≤50 years)	(>50 years)	(≤50 years)	(>50 years)
*N*	8	23	8	20
Time to SM (min)^a^	167 ± 40	248 ± 77^*^	335 ± 120	297 ± 79
Urine volume (mL)^b^	299 ± 117	353 ± 104	276 ± 139	345 ± 115
Infusion volume (mL)^b^	1613 ± 158	1637 ± 302	1731 ± 281	1612 ± 263

Values are given as mean ± standard deviation. ^a^Difference was analyzed by two-sample *t*-test between the two groups; ^b^Difference was analyzed by Mann-Whitney *U* test between the two groups.  ^*^ *P* < 0.001 versus younger age group. EA: electroacupuncture; SM: spontaneous micturition.

**Table 4:** Time to spontaneous micturition, urine volume, and infusion volume in different gender group.

	EA group		Control group	
	Female	Male	Female	Male
*N*	21	10	20	8
Time to SM (min)^a^	216 ± 85	251 ± 58	298 ± 92	335 ± 91
Urine volume (mL)^b^	333 ± 111	351 ± 106	314 ± 122	355 ± 133
Infusion volume (mL)^b^	1667 ± 309	1555 ± 144	1578 ± 220	1818 ± 316

Values are given as mean ± standard deviation. ^a^Difference was analyzed by two-sample *t*-test between the two groups; ^b^Difference was analyzed by Mann-Whitney *U* test between the two groups. EA: electroacupuncture; SM: spontaneous micturition.
